# Fates of slurry-nitrogen applied to mountain grasslands: the importance of dinitrogen emissions versus plant N uptake

**DOI:** 10.1007/s00374-024-01826-9

**Published:** 2024-05-10

**Authors:** Michael Dannenmann, Irina Yankelzon, Svenja Wähling, Elisabeth Ramm, Mirella Schreiber, Ulrike Ostler, Marcus Schlingmann, Claus Florian Stange, Ralf Kiese, Klaus Butterbach-Bahl, Johannes Friedl, Clemens Scheer

**Affiliations:** 1 Institute of Meteorology and Climate Research, Karlsruhe Institute of Technology (KIT), Atmospheric Environmental Research (IMK-IFU), Kreuzeckbahnstraße 19, 82467 Garmisch-Partenkirchen, Germany; 2 Grassland Division, Landwirtschaftliches Zentrum Baden-Württemberg (LAZBW), Lehmgrubenweg 5, 88326 Aulendorf, Germany; 3 Federal Institute for Geosciences and Natural Resources (BGR), Stilleweg 2, 30655 Hannover, Germany; 4 Land-CRAFT, Department of Agroecology, University of Aarhus, Ole Worms Allé 3, Bld. 1171, 8000 Aarhus, Denmark; 5 Department of Forest and Soil Sciences, Institute of Soil Research, University of Natural Resources and Life Sciences, Vienna, Austria

**Keywords:** ^15^N gas flux method, Dinitrogen emissions, Nitrous oxide emissions, Nitrogen balance, Plant nitrogen uptake, Organic fertilization

## Abstract

Intensive fertilization of grasslands with cattle slurry can cause high environmental nitrogen (N) losses in form of ammonia (NH_3_), nitrous oxide (N_2_O), and nitrate (NO_3_^−^) leaching. Still, knowledge on short-term fertilizer N partitioning between plants and dinitrogen (N_2_) emissions is lacking. Therefore, we applied highly ^15^N-enriched cattle slurry (97 kg N ha^−1^) to pre-alpine grassland field mesocosms. We traced the slurry ^15^N in the plant-soil system and to denitrification losses (N_2_, N_2_O) over 29 days in high temporal resolution. Gaseous ammonia (NH_3_), N_2_ as well N_2_O losses at about 20 kg N ha^−1^ were observed only within the first 3 days after fertilization and were dominated by NH_3_. Nitrous oxide emissions (0.1 kg N ha^−1^) were negligible, while N_2_ emissions accounted for 3 kg of fertilizer N ha^−1^. The relatively low denitrification losses can be explained by the rapid plant uptake of fertilizer N, particularly from 0–4 cm depth, with plant N uptake exceeding denitrification N losses by an order of magnitude already after 3 days. After 17 days, total aboveground plant N uptake reached 100 kg N ha^−1^, with 33% of N derived from the applied N fertilizer. Half of the fertilizer N was found in above and belowground biomass, while at about 25% was recovered in the soil and 25% was lost, mainly in form of gaseous emissions, with minor N leaching. Overall, this study shows that plant N uptake plays a dominant role in controlling denitrification losses at high N application rates in pre-alpine grassland soils.

**Supplementary information:**

The online version contains supplementary material available at 10.1007/s00374-024-01826-9.

## Introduction

Liquid cattle slurry has been increasingly replacing traditional fertilization with farmyard manure in mountain grasslands in Central Europe (Capriel 2013). It is well known that the use of cattle slurry can involve high gaseous and hydrological losses (Schröder et al. 2005), in particular in the form of ammonia (NH_3_) (Nyameasem et al. 2022) and nitrate (NO_3_^−^) (Chang and Entz 1996), and therefore, a relatively low plant N use efficiency and N sequestration in soils (Schlingmann et al. 2020; Schreiber et al. 2023; Zistl-Schlingmann et al. 2020). The overall high fertilizer N losses in grasslands therefore promote soil N mining, especially in organic matter-rich soils, where plant N nutrition is largely based on N from soil organic matter mineralization rather than from fertilizer (Schlingmann et al. 2020; Schreiber et al. 2023; Zistl-Schlingmann et al. 2020). The N losses impact air quality, human health, and biodiversity through NH_3_ emissions and N deposition, while NO_3_^−^ leaching threatens groundwater quality (Fangueiro et al. 2014; Fu et al. 2017; Smith et al. 2002; Sutton et al. 2008). Slurry application also leads to nitrous oxide (N_2_O) emissions (Cardenas et al. 2019; Nyameasem et al. 2022; Rodhe et al. 2006), which are lower in calcareous pre-alpine soils due to pH-induced reduction of N_2_O to dinitrogen (N_2_) (Chen et al. 2015; Malique et al. 2021; Wu et al. 2020; Zistl-Schlingmann et al. 2019). In contrast to the potent greenhouse gas N_2_O, N_2_ emissions are environmentally benign (Butterbach-Bahl et al. 2013), but still denote fertilizer loss, impact grassland N balance and potentially accelerate soil N mining (Schlingmann et al. 2020; Wu et al. 2020; Zistl-Schlingmann et al. 2019, 2020). Furthermore, from a farmer's perspective, these emissions also translate into a financial loss, highlighting the economic impact alongside ecological concerns (Laborde et al. 2021). However, N_2_ emissions remain the greatest uncertainty in ecosystem nitrogen balances (Almaraz et al. 2020), particularly in grasslands (Zistl-Schlingmann et al. 2019). This is due to notorious challenges in measuring N_2_ emissions under real-world conditions (Friedl et al. 2020; Groffman 2012).

Buchen-Tschiskale et al. (2023) applied a ^15^N gas flux (^15^NGF) method under field conditions and reported N_2_ emissions to amount to up to 3 kg N ha^−1^ after addition of 67 kg N ha^−1^ of cattle slurry via different application techniques to winter wheat mesocosms from Southern Germany. Much higher N_2_ emissions of 11–19 kg N ha^−1^ were reported based on ^15^NGF measurements in grassland in Northern Ireland after the application of cattle slurry combined with large NO_3_^−^ applications (together 65 kg N ha^−1^) (McGeough et al. 2012). In the latter studies, the addition of ^15^NO_3_^−^ combined with the labile carbon (C) in cattle slurry might have stimulated N_2_ emissions. In contrast, in the study of Zistl-Schlingmann et al. (2019), all gaseous N losses were measured from intact grassland mesocosms after the addition of 51 kg N ha^−1^ of cattle slurry, with no further ^15^N addition needed, as N_2_ losses were directly measured in an artificial N_2_-free atmosphere established with the Helium substitution soil core technique. The latter study revealed total gaseous N losses equal to half of the slurry N addition. Thereby, N_2_ emissions with 31–42% of applied slurry-N were dominating N losses and even exceeded NH_3_ losses. In this study, however, plant N uptake of N was excluded due to dark chamber measurements, which might have led to an overestimation of denitrification due to impaired plant competition for N. This appears particularly important in temperate grasslands, which are rich in soil organic matter and show enormous productivity and plant N uptake, often even quantitatively exceeding N addition by fertilization (Fu et al. 2017). Despite such findings, which at first glance seem to indicate a high fertilizer N use efficiency by plants, several recent studies tracing ^15^N from labeled organic fertilizers into plant biomass in different pre-alpine grassland soils under different manure application techniques show the opposite. These studies found low fertilizer ^15^N recovery of only 3–38% in harvested plant biomass N 1–2 months after fertilization, indicating that N sources other than fertilizer contribute up to more than 90% of plant N uptake (Schlingmann et al. 2020; Schreiber et al. 2023; Zistl-Schlingmann et al. 2020). Also, various other ^15^N tracing studies in grasslands (Bardgett and Wardle 2003; Christian et al. 1997; Harrison et al. 2007, 2008; Hart et al. 1986; Rowlings et al. 2016; Scheiner et al. 2002; Xu et al. 2010) show high variance in total ^15^N recovery in the plant biomass, with recovery rates ranging from 2 to 50%.

Ammonium (NH_4_^+^) applied via organic fertilizer to grassland soils is subject to competition by plant uptake and microbial utilization such as nitrification, with the produced NO_3_^−^ again either being taken up by plants or used by microbial processes such as denitrification (Butterbach-Bahl et al. 2013; Gao et al. 2023; Harrison et al. 2008). Hence, understanding the distribution of manure N between inorganic and organic forms, and how these forms are utilized by plants and microbes—referred to as manure N partitioning—is crucial to reducing N losses in the form of N_2_O and N_2_ and improving manure N use efficiency. In this context, a number of the aforementioned studies have investigated the fate of manure N using stable isotope techniques on time scales of months to years, and gaseous emissions of NH_3_ and N_2_O have also been increasingly well constrained. In contrast, N_2_ emissions from organic grassland fertilization have only been directly measured by a few studies, with very contrasting results (Friedl et al. 2017; McGeough et al. 2012; Zistl-Schlingmann et al. 2019). Furthermore, to our knowledge, no study has investigated the short-term partitioning of manure N in grasslands between plant N uptake and N_2_ emissions at shorter time scales relevant for a better understanding of the dynamics and magnitude of N_2_ emissions, i.e., a few days to weeks after fertilization. Given the potentially critical role of N_2_ in grassland N balances, these knowledge gaps severely hamper the development of management options aimed at reducing grassland N losses. Furthermore, knowledge of the quantitative role of plant N uptake versus N_2_ emissions will help to assess the extent to which dark chamber measurements of N_2_ emissions, as typically performed with the He soil core method, are biased by inactive grassland plants. Therefore, in this study, we applied highly ^15^N-enriched cattle slurry to pre-alpine grassland field mesocosms and followed the ^15^N signal with high temporal resolution in plant above and below ground biomass (AGB/BGB) and different vertical soil layers (temporal resolution: days 1, 3, 10, 17, 29 after fertilization). This was accompanied by direct measurements of gaseous N_2_ (using the ^15^NGF method) and N_2_O losses in daily temporal resolution, and the quantification of leached ^15^N after 29 days, allowing the calculation of a complete fertilizer ^15^N balance. Following this approach, the goals of the present study were to 1) assess the short-term dynamics of the partitioning of cattle slurry N in the plant-soil-system over 1 month in high temporal resolution; and 2) to quantify the role of N_2_ emissions versus plant N uptake in the fertilizer N balance after manure application under real field conditions. We hypothesized that a large short-term pulse of N_2_ emissions would substantially contribute to total gaseous N losses, potentially surpassing plant N uptake during the first days following fertilizer application. For longer time scales of weeks to one month after fertilization we expected plant N uptake of fertilizer-derived N to increase in importance compared to soil-derived N, thereby more and more exceeding fertilizer-derived N_2_ losses.

## Material and methods

### Study sites and experimental design

**Fig. 1:**
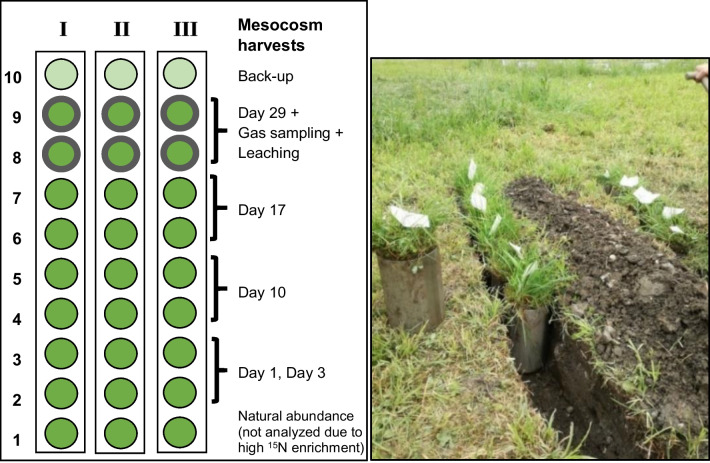
Experimental design and sampling scheme of mesocosms. Please note that I, II, III represent different sampling plots at the site of soil origin. Resin bags for ^15^N analysis in leached NH_4_^+^ and NO_3_^−^ were installed under the six mesocosms that were harvested at the end of the experiment. These mesocosms were also used for measurements of gaseous losses of N_2_O and N_2_ using static chambers over the entire time span of the experiment

This study was conducted in the grasslands of the TERENO pre-alpine observatory in Southern Germany (Kiese et al. 2018). Sites are located in flat valley bottoms of the Northern Calcareous Alps. In total 30 intact plant-soil mesocosms of 16 cm diameter and 25 cm depth were excavated at three replicated plots (10 per plot) at the Graswang site (860 m a.s.l., (47°34′10.2’’N, 11°01′54.1’’E) in August 2016 and re-buried in situ within the Graswang site. The sampling site has a mean annual temperature (MAT) of 6.9 °C and mean annual precipitation (MAP) of 1347 mm (2014—2017) (Zistl-Schlingmann et al. 2020). The soil, characterized as a C- and N-rich Haplic Cambisol derived from limestone and dolomite, comprises 51% clay, 40% silt, and 9% sand (Kiese et al. 2018). Based on the mesocosm analyses of this study, bulk density was 0.60 ± 0.03 g cm^−3^ (0–4 cm); 0.64 ± 0.02 g cm^−3^ (4–10 cm) and 0.91 ± 0.02 g cm^−3^ (10–25 cm) (Garcia-Franco et al. 2024). The soil organic carbon (SOC) and total N (TN) content of the soil is 118 ± 10 and 12 ± 1 mg g^−1^ and (0–5 cm depth), and 98 ± 9 and 11 ± 1 mg g^−1^ (5–15 cm depth) (Garcia-Franco et al. 2024). Due to the carbonate buffer system, pH was measured at 7 in the 0–15 cm depth range (Unteregelsbacher et al. 2013). Despite large gross N mineralization and nitrification rates, both soil NH_4_^+^ and NO_3_^−^ concentrations remain below 10 mg N kg^−1^ sdw throughout the growing season when there is no fertilization (Wang et al. 2016). The vegetation is dominated by the grasses *Trisetetum flavescentis*, *Dactylis glomerata*, *Festuca pratensis* und *Festuca rubra* and the herbaceous plants like *Pimpinella major*, *Plantago lanceolata* und *Trifolium pratense* (Schlingmann 2020). After four years of installation at the sampling site Graswang, for logistical reasons mesocosms were translocated on June 4, 2020, to a similar grassland site at Garmisch-Partenkirchen (730 m asl, close to the Alpine Campus of KIT, where MAT and MAP were 8.4 °C and 1360 mm, respectively (2014–2017, DWD). The experiment of this study started on June 22, 2020, with the application of ^15^N labeled slurry to all mesocosms. The experimental design is illustrated in Fig. 1. At days 3, 10, 17 and 29 after fertilization, entire mesocosms (N = 6 each) were destructively harvested to determine ^15^N excess recovery from the labeled slurry. Because of logistic and time constraints, the first harvest on day 1 after fertilization was a mesocosm subharvest of the day 3 cores. For this, we used a corer of 5 cm diameter (0–4 cm depth, and AGB) and 3 cm diameter (4–10 cm and 10–25 cm). Hence, each of the five harvests was followed by AGB ang BGB sampling, and soil sampling in 0–4, 4–10, and 10–25 cm depths as described below. Day 3 sampling was corrected for the missing soil mass and plant biomass due to the subsampling at day 1. Following this approach, data on soil and plant N pools and ^15^N recovery were obtained for days 1, 3, 10, 17 and 29 after slurry application.

### ^15^N labeled cattle slurry

The ^15^N labeling of the mesocosms was conducted via the application of ^15^N enriched liquid cattle slurry. The fresh slurry originated from a local farmer and was analyzed for N compounds by a commercial laboratory (Raiffeisen-Laborservice, Ormont, Germany). The analysis of N compounds was conducted following international standards for N analysis. Total nitrogen (TN) and dissolved N was determined by elemental analysis as outlined in DIN ISO 13878 (DIN 1998). Organic N was calculated based on the difference between total and dissolved N. The slurry had a pH of 7.9 and was characterized by a low background N content of 1.4 g N kg^−1^ FW (69.7 g N kg^−1^ DWa), with 0.4 g N kg^−1^ FW consisting of organic N and 1 g N kg^−1^ FW consisting of dissolved N, i.e., NH_4_^+^-N and urea-N. This low N slurry was ^15^N-labeled by adding 2.2 g urea-N g^−1^ FW and 2.2 g NH_4_^+^-N g^−1^ FW at 99 atom% ^15^N enrichment. For this, we generally followed the ^15^N slurry labeling procedure described in detail by Schlingmann et al. (2020), but used higher ^15^N addition rates to achieve the required high ^15^N tracer enrichments for the detection of ^15^N_2_ formation through denitrification (Friedl et al. 2020). Immediately prior to fertilization of the mesocosms, the ^15^N tracer was added to the slurry in a glass bottle and vigorously mixed. Following the regional standard farming practice of surface spreading, we then applied the slurry at a rate of 1.8 m3 ha^−1^ which translates to 40 ml for each mesocosm (Schlingmann et al. 2020). This equaled an N addition of 97.2 kg N ha^−1^, strongly dominated by plant-available N (urea- and NH_4_^+^-N). Through this procedure, 175.9 mg ^15^N in excess of natural abundance was added per mesocosm (79 atom % ^15^N enrichment). One hour following slurry application, we simulated a precipitation event of 30 mm, applied in 6 doses within 3 h.

### Mesocosm harvest, soil and plant sampling

We followed the mesocosm sampling scheme described in detail by Schlingmann et al. (2020) and Schreiber et al. (2023, see also supplementary information), with the complete harvest being conducted within one day. In short, the soil column was pushed out of the stainless-steel core with a manual hydraulic device (Schreiber et al. 2023). Aboveground biomass was then sampled by cutting the turf directly above the soil with scissors. Subsequently, the soil column was cut into 3 pieces with a saw: 0–4 cm, 4–10 cm, 10–25 cm depth. Total soil of each layer was homogenized by hand in a bucket for at least 10 min. Roots were hand-picked and washed with tap water (Schreiber et al. 2023). Soil, AGB and BGB samples were dried at 55° C to a constant weight directly after sample preparation. Subsequently, samples were homogenized again, and a representative subsample was ground to a fine powder with a ball mill (Retsch Schwingmühle MM2, Haan, Germany) and stored until analysis in a desiccator over silica gel. The ^15^N enrichment in total soil N, AGB and BGB samples as well as the respective TN concentration was measured by elemental analysis coupled to isotope ratio mass spectrometry (Yankelzon et al. 2024a, b). For the assessment of inorganic N, fresh soil samples underwent an extraction process using 0.5 M K_2_SO_4_, maintaining a 1:2 ratio of soil weight to extract solution volume (Dannenmann et al. 2016). Following extraction, the samples were transferred into Falcon tubes and stored in a freezer for subsequent analysis. Concentrations of dissolved NH_4_^+^-N and NO_3_^−^-N were measured colorimetrically using a microplate spectrometer (BioTek Instruments, Inc.), following the methods described by Kempers and Zweers (1986) and Pai et al. (2021). Soil extracts were also analyzed for DOC concentrations (Multi N/C 3100, Analytik Jena, Germany) according to Dannenmann et al. (2016), ^15^N enrichment in NH_4_^+^ and NO_3_^−^ was determined by sequential diffusion on acid filter traps, which were then analyzed via elemental analysis (Flash EA, Thermo Scientific, Waltham, MA, USA) coupled to isotope ratio mass spectrometry (IRMS) (Delta PlusXP, Thermo Scientific, Waltham, MA, USA) as described in detail by Schreiber et al. (2023). Gravimetric soil water content was determined by drying the fresh soil in an oven (105 °C) for over 24 h. The latter was used to calculate water-filled pore space (WFPS), thereby considering the respective mesocosm-layer specific bulk density, and a particle density of 2.65 g cm^−3^. We conducted additional two samplings for the determination of WFPS using a 3 cm thick corer on days 7 and 14.

### Nitrogen leaching

Nitrogen leaching rates were measured with the six mesocosms that were harvested at the end of the experiment (Fig. 1). The leaching of mineral N was determined from the accumulation of NH_4_^+^- and NO_3_^−^- N and -^15^N on resin bags (Dannenmann et al. 2018). For this purpose, bags containing ion‐exchange resins (45 g of Amberlite, IR 120 [Na^+^‐ion exchanger resin] and 45 g Dowex 1 × 8 [Cl^−^‐ion exchanger resin]) were placed under six mesocosms and harvested at the end of the experiment. Resin bags were brought to the laboratory facilities of KIT-IMK-IFU in Garmisch-Partenkirchen and extracted with 1 M NaCl solution. The extracts were analyzed for NH_4_^−^-N and NO_3_^−^-N concentrations and ^15^N-enrichment in NH_4_^+^ and NO_3_^−^ using the SPINMAS technique (Stange et al. 2007). The measurements were carried out in an automated sample preparatory (SPIN unit; InProcess, Bremen, Germany) coupled with a mass spectrometer (GAM 400; InProcess, Bremen, Germany).

### Emissions of N_2_O and N_2_

Emissions of N_2_O and N_2_ were measured using the manual static chamber technique on the six mesocosms harvested on day 29. The dark steel chambers covered the entire mesocosms and had a height of 15.5 cm (see Unteregelsbacher et al. 2013 for further details). For quantification of N_2_O emissions, the sampling frequency was daily for the first two weeks post-fertilization. This frequency was then reduced to three measurements per week from the third week onwards. The chamber closure time was 120 min. Gas samples were taken between 10–12 am at 0, 30, 60 and 120 min after chamber closure and then transferred into pre-evacuated 12 ml glass vials with a double wadded Teflon/silicon septa cap (Labco, UK) and analyzed for N_2_O concentrations using gas chromatograph connected to an autosampler (SRI 8610C, SRI Instruments, Torrance, USA), following the method described by Rehschuh et al. (2019). Nitrous oxide fluxes were calculated based on the slope of linear increase in N_2_O concentration over time, thereby considering air temperature and atmospheric pressure (Rehschuh et al. 2019). Quality control involved determining the minimum detectable concentrations. This was done by multiplying the standard deviation of ambient air N_2_O levels by the t-value for a 95% confidence interval. These concentrations were then translated into fluxes, setting a threshold below 0.4 g N ha^−1^ day^−1^.

The ^15^NGF method was applied to quantify N_2_ losses daily for the first 7 days following fertilization, and then every two to four days until day 29. For this, additional vials were filled simultaneously with the N_2_O sampling, at 0, 30 and 120 min after chamber closure. These vials were analyzed for N_2_O and N_2_ and their respective isotopologues ((^15^N^14^N, ^15^N^15^N; [^14^N^15^N^16^O + ^15^N^14^N^16^O] and ^15^N^15^N^16^O) using an isotope ratio mass spectrometer (IRMS) (Sercon Limited, 20–20, UK) linked to a Sercon Cryoprep trace gas concentration system.

Emissions of N_2_ were calculated using the ^15^NGF method (Friedl et al. 2020). Briefly, measurements of the ion currents (I) via IRMS at the mass-to-charge ratio (m/z) 44, 45, and 46 allowed the molecular ratios ^45^R (^45^I/^44^I) and ^46^R (^46^I/^44^I) to be calculated for N_2_O. The ^15^N enrichment of the soil NO_3_^−^ pool undergoing denitrification (*a*D) (Fig. S1) was estimated using ^45^R and ^46^R. The I at m/z 28, 29 and 30 gave ^29^R (^28^I/^29^I) and ^30^R (^28^I/^30^I) for N_2_, and the change in the respective ratios during chamber closure was expressed as Δ^29^R and Δ^30^R. Assuming that N_2_O derived from denitrification, and N_2_ were produced from the same NO_3_^−^ pool, fluxes of N_2_ were estimated using Δ^30^R and *a*D (Mulvaney 1984; Stevens and Laughlin 2001), corrected for air temperature, and air pressure and expressed in g ha^−1^ day^−1^. The precision of the IMRS determined prior analysis at 95% confidence intervals (n = 10) was 6.16 × 10^–7^ and 4.60 × 10^–7^ for ^29^R and Δ^30^R, respectively. The excess ^15^N enrichment of N_2_O was used to calculate its fertilizer-derived fraction as outlined in Takeda et al. (2022). The measured %^15^N excess in N_2_O was corrected to account for the N_2_O existing in the headspace.

### Calculation of ^15^N recovery in plant and soil N, and leached N

The excess ^15^N amount (mg) in all investigated N pools was calculated using the following equation.

$${N}_{pool}*\left(\frac{{15N}_{L}- 0.3663}{100}\right)$$

where *N*_*pool*_ is the amount of N [mg N mesocosm^−1^] in the plant or depth-specific soil N pool and *15N*_*L*_ is the enrichment (atom% ^15^N) of the respective N pool. We used 0.3663% as the natural abundance of ^15^N; errors induced by possible slight variations of ^15^N natural abundance were negligible due to the high enrichment obtained from the very large ^15^N slurry labeling. Dividing ^15^N excess amount in the analyzed pools by the cumulative ^15^N excess addition through slurry fertilization at the sampling time (175.87 mg ^15^N excess per mesocosm) revealed the ^15^N excess recovery, expressed as a percentage.

### Fertilizer N fates and balance

The calculation of fertilizer N balances followed the approach described by Yankelzon et al. (2024a, this issue). Specifically, N flows from fertilizer into total soil N, into leached N, as well as into plant AGB and BGB N were calculated by dividing the respective ^15^N excess recovery rate (% of applied fertilizer ^15^N excess) by 100 and multiplying with the amount of total added fertilizer N (97.2 kg N ha^−1^). This total slurry N amount also includes unlabeled polymeric organic N of 7 kg N ha^−1^, which might behave differently compared to the plant available N which was ^15^N labeled. Unrecovered ^15^N was assumed to equal total gaseous N losses. Subtracting measured N_2_O and fertilizer-derived N_2_ losses then revealed NH_3_ emissions, assuming negligible NO emissions as confirmed previously for similar grassland mesocosms (Zistl-Schlingmann et al. 2019).

### Statistical analysis

There were six replicate mesocosms per sampling date. Statistical analyses were performed in R version 4.1.3 with a 5% significance level. Before applying parametric tests, it was assured that data were normally distributed with Shapiro–Wilk tests or that variance was homogenous with Levene’s tests. Non-normally distributed data were log-transformed. We performed ANOVAs including post-hoc Tukey analyses correcting for multiple testing.

## Results

### Meteorological data

**Fig. 2:**
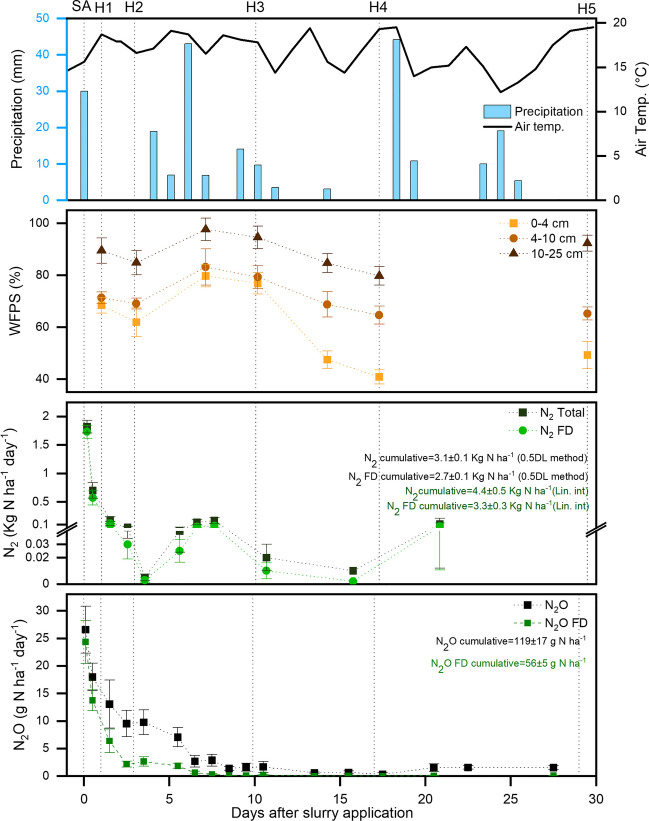
Daily precipitation and mean air temperature during the experiment (A); soil water-filled pore space (WFPS) in the three sampled soil layers (B); N_2_ emissions (C); and N_2_O emissions (D). Note that in (C and D) both total N emissions and fertilizer-derived (FD) emissions are provided. Furthermore, for N_2_ two interpolation techniques were used (linear interpolation between actual measurements; and a more conservative interpolation using N_2_ emissions of half of the method detection limit for all days with no measurements or measurements with no detectable N_2_ fluxes. N_2_O cumulative fluxes were based on linear interpolation. Slurry application (SA) and harvest times (H1-H5) are indicated by dotted lines. The error bars depict the standard errors derived from the means of six replicates

Following the slurry application on June 22, we directly simulated rainfall of 30 mm (Fig. 2A). There was no natural precipitation over three days after fertilization. However, in the following three weeks, precipitation was observed during 13 days, with the daily sum even exceeding 40 mm on June 28th and July 10th. Consequently, this resulted in high values of WFPS up to 100% in the deepest soil layer, with WFPS generally increasing with soil depth (Fig. 2B). In the very wet first 10 days of the experiment, also topsoil WFPS (0–4 cm and 4–10 cm) was high (range 60–80%). In all soil layers, soil moisture reached its highest values on day 8, induced by a daily precipitation of > 40 mm on day 7 (Fig. 2B). After these wet first 10 days, WFPS in all depths decreased until day 17, but only WFPS in 0–4 cm declined to ca 40% WFPS, while it did not drop below 60% WFPS in 4–10 cm (Fig. 2B). The presumable increase in WFPS after the second large precipitation event > 40 mm on June 10 was not captured by the sampling design (Fig. 2B). Daily mean air temperatures were quite variable during the course of the experiment and oscillated between ca 12 °C and 20 °C.

### Emissions of N_2_ and N_2_O

The highest N_2_ emissions were measured immediately after fertilizer application, totaling 1.8 kg N ha^−1^ day^−1^, thereof 1.7 kg N ha^−1^ day^−1^ derived from fertilizer (Fig. 2C). These emissions quickly declined to values close to zero within 3 days, and slightly increased again after the large precipitation event on day 7 to form a smaller second peak of N_2_ emissions (Fig. 2B, C). This smaller peak slowly declined afterward, thereby following the dynamics of WFPS (Fig. 2B, C). The sampling on day 21 also revealed relatively high N_2_ emissions, which occurred two days after the second heavy precipitation event (Fig. 2A, C). Cumulative total N_2_ emissions were 4.4 kg N ha^−1^ when linear interpolation between measurement days was applied, and 3.1 kg N ha^−1^ when N_2_ fluxes at days with no measurements or undetectable fluxes were set to 50% of the method detection limit (conservative estimate). For fertilizer-derived N_2_ emissions, these values amounted to 3.3 and 2.7 kg N ha^−1^, respectively.

Nitrous oxide emission followed a generally similar pattern compared to N_2_ emission, but with a slower decline of the emissions (Fig. 2D). The highest emission, 27 g N ha^−1^ day^−1^ (24 g N ha^−1^ from fertilizer-derived N_2_O), was observed on the day of fertilization, followed by a subsequent decline to low background fluxes approximately within one week. Subsequently, only very minor changes in N_2_O fluxes were observed. The cumulative N_2_O fluxes totaled 0.1 kg N ha^−1^, of which 0.06 kg N ha^−1^ were fertilizer-derived. The N_2_O:(N_2_O + N_2_) emission ratio (R_N2O_) was well below 0.1 throughout the course of the measurements, except for days 3–6, when N_2_ emissions almost had disappeared, but minor N_2_O emissions still were present (Fig. S2). The overall R_N2O_, however, was very low, at 0.03.

### Soil ammonium, nitrate and dissolved organic carbon

**Fig. 3:**
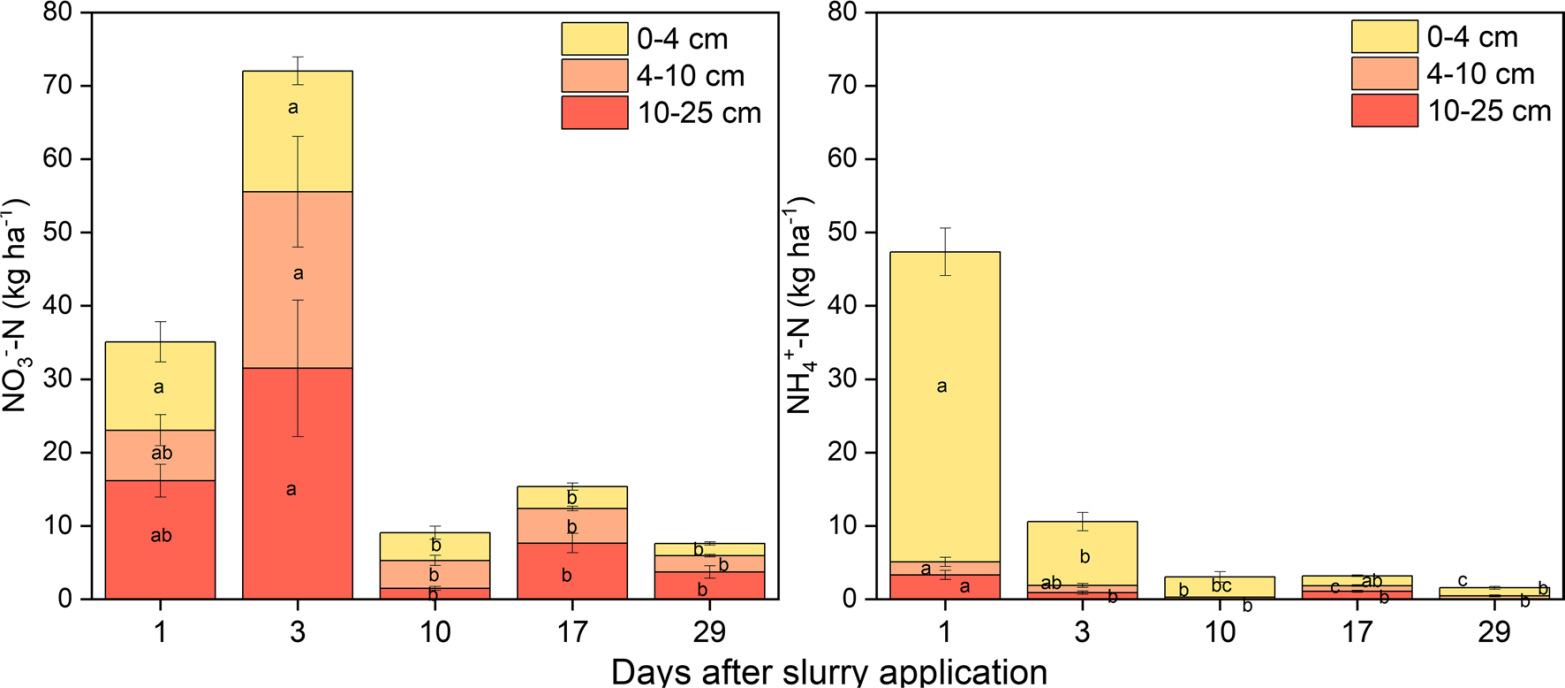
Soil NO_3_^−^ and NH_4_^+^ stocks during the experiment. Uncertainty is represented by the standard error calculated from the mean of six replicates. Different indices indicate significant changes across different sampling times in different depths

One day after fertilization, we found 47 kg NH_4_^+^-N in the soil of the mesocosms, thereof 42 kg NH_4_^+^-N in 0–4 cm depth (Fig. 3). Nitrate–N amounted to 35 kg N ha^−1^ and was more evenly spread across the three soil layers. Accordingly, the sum of mineral N found in all soil layers sampled (82 kg N ha^−1^) was close to the total added fertilizer N of 97 kg ha^−1^, of which 90 kg N ha^−1^ was in dissolved form (mainly urea and NH_4_^+^). On day 3 after fertilization, we still found a total of 82 kg N ha^−1^ of mineral N in the soil (Fig. 3), with NH_4_^+^ contributing only 10 kg N ha^−1^ and NO_3_^−^ having doubled to 72 kg N ha^−1^ (Fig. 2). On days 10 and 17 after fertilization, the mineral N stocks in the soil had decreased by almost one order of magnitude to about 15 kg N ha^−1^, and further decreased to about 10 kg N ha^−1^ on day 29 at the end of the experiment (Fig. 3), with NO_3_^−^ N always dominating the mineral N stocks during this time. Dissolved organic carbon (DOC) stocks dropped between days 1 and 3 and then remained unchanged (Fig. S4). The DOC: NO_3_^−^ ratios and in particular the DOC: dissolved mineral N ratios in the first 3 days of the experiment were lowest in the top 0–4 cm (Fig. S5, S6). Specifically, DOC: dissolved mineral N ratios in the top 4 cm were as low as two on day 1 and only increased to about four (day 3) and five (day 10) (Fig. S6).

### ^15^N fertilizer recovery

**Fig. 4:**
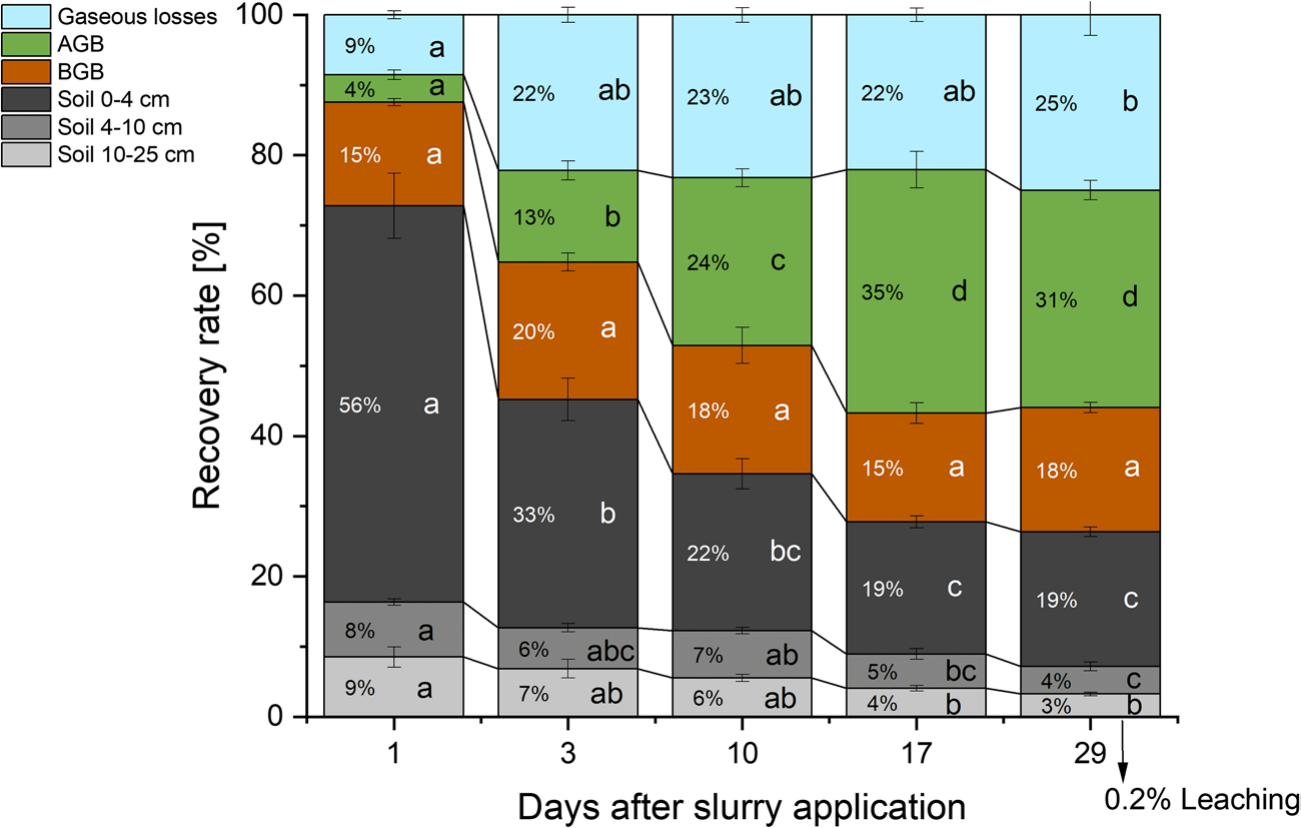
Recovery of fertilizer ^15^N excess in aboveground (AGB) and belowground (BGB) plant biomass, and in soil. Unrecovered ^15^N was assumed to equal total gaseous N losses (NH_3_, N_2_O, N_2_). Leaching (0.2% recovery only) was measured only for the last sampling on day 29. The error bars illustrate the standard errors obtained from averaging six replicates

One day after fertilizer application, we recovered 91 ± 5% of the added ^15^N-labeled fertilizer in the plant-soil system, indicating an initial gaseous loss of 9% during the first day (Fig. 4). The total ^15^N recovery decreased to a value of 78 ± 3% by day 3, but then did not change significantly until the end of the experiment, when the recovery was still 75 ± 2% (Fig. 4). Considering the negligible cumulative ^15^N recovery in leached N of 0.2% at the end of the experiment, this implies that the losses of about 25% were mainly via gaseous pathways (Fig. 4).

At day 1, we already recovered 15% of the tracer in the roots (Fig. 4). This ^15^N recovery in the roots did not significantly change in the further course of the experiment (Fig. 4). With on average 90% relative contribution, roots in 0–4 cm depth were most important for ^15^N excess recovery, while roots in 4–10 cm depth (7% contribution) and in 10–25 cm depth (3%) only contributed to a minor extent to the tracer recovery (data not shown). With only 4% we recovered almost four times less ^15^N tracer in AGB compared to roots one day after the application of ^15^N fertilizer. However, the ^15^N recovery in AGB quickly increased to reach its maximum of 35% at day 17 with no further increase to the end of the experiment (Fig. 4). At days 17 and 29, about half of the ^15^N tracer was recovered in AGB and BGB.

We recovered almost 75% of fertilizer ^15^N excess in soil, one day after application, mostly in 4–10 cm, and to a much smaller extent also in 4–10 and 10–25 cm depth (Fig. 4). At day 3, ^15^N recovery in NO_3_^−^ exceeded ^15^N recovery in NH_4_^+^ by already more than a factor of six (Fig. S3). In the following days and weeks, ^15^N recovery decreased in all soil layers, but particularly in 0–4 cm depth, until day 17 to about 25% of the applied ^15^N excess (Fig. 4). This equaled the increasing ^15^N excess recovery in plant biomass. From day 17 to day 29, the ^15^N excess recovery in soil did not significantly change, as was also observed for ^15^N recovery in plant biomass. The ^15^N recovery balance over time indicated that there were soil-born gaseous ^15^N losses between days 1 and 3 (Fig. 4). However, subsequent decreases in soil ^15^N recovery were matched by parallel increases in AGB ^15^N recovery, while no significant increase in unrecovered ^15^N excess (i.e., gaseous losses) was observed (Fig. 4).

### Aboveground biomass N accumulation and origin

The AGB increased from 1.6 ± 0.3 t DM ha^−1^ the day after fertilization to 4.9 ± 0.4 t DM ha^−1^ on day 10, but did not show further significant changes between day 10 and day 29 (Table 1). Aboveground plant N increased from initially 24.6 ± 4.0 kg N ha^−1^ on day 1 after fertilization to reach its maximum of very large 100.4 ± 5.6 kg N ha^−1^ (equaling total fertilizer N without fertilizer N losses) at day 17 after fertilization, with no further increase being observed until day 29 (Table 1). Already one day after fertilization, 3.8 ± 0.7 kg N ha^−1^ or 15.3% of total AGB—N was derived from fertilizer. At day 3, this fertilizer-derived N in AGB amount was tripled, and reached its maximum of 33.6 ± 2.5 kg N ha^−1^ 17 days after fertilization. Still, N from recent fertilizer application was of minor importance compared to other plant N sources, with fertilizer N contribution to AGB-N of 13–15% in the first three days and 32–34% during the later sampling times (Table 1). Fertilizer-derived N in total BGB was as high as 14 kg N ha^−1^ one day after slurry application, but then did not further increase (Table 1). Consequently, after 10 days, there was always more fertilizer-N in AGB compared to BGB.

**Table 1:** Temporal (days after slurry application and harvest (H) numbers) dynamics of aboveground plant biomass (expressed as dry matter, DM) and its N uptake. The contribution of fertilizer-derived N to total belowground biomass N is also given. Uncertainty is expressed as the standard error of the mean. Different indices indicate statistically significant changes over time

**Days after slurry application**	1 (H1)	3 (H2)	10 (H3)	17 (H4)	29 (H5)
Aboveground plant biomass (t DM ha^−1^)	1.6 ± 0.3 a	2.7 ± 0.3 b	4.9 ± 0.3 c	4.3 ± 0.1 bc	4.9 ± 0.4 c
Total aboveground plant N (kg N ha^−1^)	24.6 ± 4.0 a	49.3 ± 5.3 b	73.1 ± 1.3 bc	100.4 ± 5.6 c	95.6 ± 3.8 c
Fertilizer N in aboveground biomass (kg N ha^−1^)	3.8 ± 0.7 a	12.6 ± 1.3 b	23.2 ± 1.2 c	33.6 ± 2.5 c	30.0 ± 1.2 c
Fertilizer N in aboveground biomass (%)	15.3 ± 1.0 a	12.6 ± 1.3 b	31.6 ± 1.3 bc	33.5 ± 1.7 c	31.6 ± 1.6 bc
Fertilizer N in belowground biomass (kg N ha^−1^)	14.4 ± 0.5 a	19 ± 1.2 a	17.8 ± 2.5 a	15.1 ± 1.4 a	17.2 ± 0.7 a

### Fertilizer N balance after one month

Despite the aforementioned low contribution of fertilizer N to total N in harvested AGB (less than 1/3), plant N uptake accounted for about half of the fertilizer N after one month, with 31% of fertilizer being allocated to AGB and 18% to BGB (Table 2). Only about 25% of fertilizer N was still recovered in soil, while about 25% was subject to N losses (Table 2). Among the directly measured losses, fertilizer-derived N_2_ emissions accounted for 3.4% (3.3 kg N ha^−1^), followed by leached fertilizer N (0.2 kg N ha^−1^ or 0.2%) and N_2_O emissions (0.06 kg N ha^−1^ or 0.06%) (Table 2). Based on a previous study (Zistl-Schlingmann et al. 2019), nitric oxide (NO) emissions in this soil are negligible, also after fertilization. Therefore, the remaining losses of 20.7 kg N ha^−1^ or 21.3% are assumed to be attributed to NH_3_ emissions.

**Table 2:** Fertilizer N balance on day 29. AGB/BGB are aboveground/belowground plant biomass. The first column describes total N flows, while the second column provides their importance in % of fertilizer N addition

	**kg N ha**^**−1**^	**%**
**Fertilizer addition**	97.2	100
To AGB	30.1	30.9
To BGB	17.2	17.7
Soil storage (total soil N)	25.6	26.3
Leached fertilizer N	0.2	0.2
Fertilizer N_2_ emissions	3.3	3.4
Fertilizer N_2_O emissions	0.06	0.06
NH_3_ emissions (mass balance)	20.7	21.4
NO emissions	negligible	negligible

## Discussion

### Dinitrogen emissions from slurry-fertilized montane grassland

This study is one of the few reports of direct in situ measurements of N_2_ emissions from grasslands. Based on the applied ^15^NGF method, we constrained total N_2_ emissions after the application of 97.2 kg of slurry-N (thereof 90 kg in form of NH_4_^+^ and urea-N) to 3.1 to 4.4 kg N ha^−1^ depending on the interpolation approach, with 2.7 to 3.3 kg N ha^−1^ being derived from the applied fertilizer. The very low N_2_O emissions of 0.1 kg N_2_O-N ha^−1^ observed from the studied mesocosms are well in line with earlier studies (Unteregelsbacher et al. 2013; Zistl-Schlingmann et al. 2019) and result in an extraordinarily low cumulative N_2_O/(N_2_ + N_2_O) ratio (R_N2O_) of 0.03. This is related to the high pH values in the soil around 7 which promotes high N_2_O reductase activity (Chen et al. 2015; Wu et al. 2020), thereby reducing N_2_O to N_2_ in the terminal step of denitrification. Furthermore, limited diffusion capacity induced by the clayey texture and high precipitation-induced WFPS as well as high O_2_ consumption through respiration, will also promote full denitrification to the end product N_2_ (Butterbach-Bahl et al. 2013; Friedl et al. 2022).

It is noteworthy that low R_N2O_ was also observed during the first three days after fertilization, when high NO_3_^−^ concentrations coincided with WFPS of 60–70% in the topsoil (Fig. 2). Given the ecology and stoichiometry of denitrification, these conditions should have limited the use of N_2_O as an electron acceptor, resulting in higher R_N2O_ and significant N_2_O emissions. The absence of significant N_2_O emissions further supports the dominant role of pH in regulating denitrification stoichiometry in calcareous soils (Dannenmann et al. 2008; Liu et al. 2010). Still, high NO_3_^−^ concentrations might have contributed to the increasing R_N2O_ between days 3 and 6 (Fig. S2), which however was observed when N gas emissions had largely decreased. Taken together, this suggests that the addition of N-rich slurry hardly leads to increased N_2_O emissions from prealpine grassland soils.

Despite the application of very N-rich manure to soil that supports high total denitrification and N_2_ emission rates, the N_2_ emissions reported in this study were lower than in most of the few other available grassland studies. For example, McGeough et al. (2012) applied 65 kg NH_4_^+15^NO_3_^−^ to grassland in Northern Ireland and reported emissions of 16.5 kg N_2_-N ha^−1^ (R_N2O_ = 0.22) and 10.5 kg N_2_-N ha^−1^ (R_N2O_ = 0.26) when a nitrification inhibitor was added. The latter study alternatively also used slurry instead of mineral N fertilizer, amended with ^15^NO_3_^−^ (also 65 kg N ha^−1^) and then measured associated N_2_ emissions of 13.5—18.7 kg N ha^−1^ (R_N2O_ 0.31 – 0.36). Buchen et al. (2016) assessed N_2_ emissions from grassland in Germany as affected by renewal and conversion to maize cropping. These authors reported, after the application of 80 kg ^15^NO_3_^−^ ha^−1^, N_2_ emissions of 21 kg N ha^−1^ (old grassland), 12.7 kg N ha^−1^ (after grassland renewal) and 40 kg N ha^−1^ (after conversion to maize cropping), i.e., several fold larger N_2_ emissions than in our study, while the mean R_N2O_ of 0.03 was comparable. Friedl et al. (2017) added only 37 kg ^15^N-labeled urea-N ha^−1^ to irrigated perennial dairy pastures in Australia for in situ quantification of denitrification fluxes using the ^15^NGF method. Despite lower N addition, they found similar N_2_ emission rates like in the present study, ranging between 1.1—3.9 kg N ha^−1^ depending on soil type and addition of nitrification inhibitors, while N_2_O emissions with 0.03 to 0.16 kg N ha^−1^ were also very low. Finally, Zistl-Schlingmann et al. (2019), using similar soil–plant mesocosms as in this study, revealed very high N_2_ emissions with a different laboratory incubation method, i.e., the He soil core technique. In this study, 51 kg N ha^−1^ was added as unmodified cattle slurry, and N_2_ emissions with 16–21 kg N ha^−1^ dominated fertilizer N losses, followed by NH_3_ (3.5 kg N ha^−1^), N_2_O (0.2–0.5 kg N ha^−1^) and NO emissions (0–0.2 kg N ha^−1^). Hence in the study of Zistl-Schlingmann et al. (2019), N_2_ emissions were about 5–6 times larger than measured in this study, while R_N2O_ ranging from 0.01 to 0.04 was very similar. The latter study probably overestimated denitrification because the dark incubation prevented plant N uptake so that more NH_4_^+^ and NO_3_^−^ were available for denitrification. This view is strongly supported by our study, as we found uptake of fertilizer N into AGB and BGB in the first 3 days of 33 kg N ha^−1^, i.e., one order of magnitude larger than denitrification N losses. It seems very plausible that denitrification would have been significantly larger without this large plant N uptake. This suggests that incubation systems based on the He soil core technique should be built with transparent chambers and light sources to allow for plant-soil-interactions such as competition for N, despite this strongly increases the engineering efforts involved in building such extremely gastight systems operating with He/O_2_ atmosphere (see Yankelzon et al. 2024b, this issue). Schlingmann et al. (2020) conducted a field lysimeter experiment with the same soil using two applications of ^15^N labeled cattle slurry of together 76 kg N ha^−1^ and reported total seasonal gaseous N losses of 37 kg N ha^−1^ based on unrecovered fertilizer ^15^N. Referencing the relative contribution of N_2_, N_2_O, NO and NH_3_ to gaseous N losses obtained in the laboratory He soil core study of Zistl-Schlingmann et al. (2019) to the unrecovered slurry ^15^N of the lysimeter field study would result in a slightly more conservative seasonal estimate of N_2_ emissions of 28–32 kg N_2_-N ha^−1^ season^−1^ or 14–16 kg N_2_-N ha^−1^ for one fertilization event.

Nevertheless, the relatively low N_2_ emissions measured in this study under in situ conditions are surprising because the soil has a high pH, NO_3_^−^ concentration and WFPS, all of which support ideal conditions for denitrification. However, denitrification as a heterotrophic process is also highly dependent on labile C sources. Therefore, low denitrification rates under high NO_3_^−^ availability and WFPS in this study could be due to a lack of labile C, which is confirmed by the very low DOC: mineral N ratios in the topsoil (Fig. S6). Furthermore, the huge conversion of NH_4_^+^ to NO_3_^−^ of about 36 kg N ha^−1^ between day 1 and day 3, suggesting net autotrophic nitrification of 18 kg N ha^−1^ day^−1^ also supports a lack of available C. It has already been shown that autotrophic nitrification dominates gross nitrification (Wang et al. 2014), and these net rates are very high given a reported cumulative growing season gross nitrification of only ca 200–250 kg N ha^−1^ season^−1^ in the studied soil (Wang et al. 2016). Because autotrophic nitrification is a competitive inferior process in NH_4_^+^ partitioning compared to the energetically superior heterotrophic microbial NH_4_^+^ immobilization, it only occurs at high rates under limited labile C availability (Butterbach-Bahl and Dannenmann 2012). Therefore, the very high ammoniacal N application could have initially triggered a very short-lived growth of free-living heterotrophic microorganisms until the labile C dissolved in the soil was exhausted, which could then also have impaired denitrification, thus explaining the rapidly declining N_2_ emissions. This view is also supported by the relatively low soil N stabilization in this study compared to a previous study, which found a much larger soil ^15^N recovery driven by microbial immobilization (Zistl-Schlingmann et al. 2020), despite this comparison is only possible for a period of 1 month.

However, also method-inherent shortcomings of the ^15^NGF method might lead to an underestimation of N_2_ emissions. While the He soil core technique quantifies all N_2_ emissions independent of their origin in the soil profile and independent of the source process and probably overestimates emissions from plant-soil systems in dark chambers as outlined above, the ^15^NGF technique might systematically underestimate N_2_ emissions. This is mainly because of heterogeneous tracer application, and due to ^15^N_2_ subsoil diffusion and storage (Arah 1992; Friedl et al. 2020; Micucci et al. 2023; Vanden Heuvel et al. 1988; Well et al. 2019). With a ^15^N tracer recovery of 56% observed one day after the addition in the 0–4 cm soil depth and 17% in the 4–25 cm soil depth, our study highlights the difficulty of homogeneously labeling vertical soil profiles (Fig. 3). Only N_2_ from unlabeled N sources which are well mixed with labeled N will be detected as ^15^N_2_ flux in the chamber headspace. It thus appears possible that we missed N_2_ emissions from deeper soil layers, where there was not sufficient mixing with the ^15^N tracer. However, this should be of little relevance for fertilizer-derived N_2_ emissions. The underestimation due to heterogenous ^15^N labeling has been estimated to be around 25% (Arah 1992; Vanden Heuvel et al. 1988), and an even larger underestimation due to subsoil diffusion of up to 70% was reported by Well et al. (2019). Consequently, N_2_ emissions could be up to twice as high as suggested by our direct measurements using the ^15^NGF method, i.e., about 6 kg N for a single manure application, which would be closer to the above estimate of 14–16 kg derived from the combination of laboratory measurements with the He technique and field ^15^N balance studies of Zistl-Schlingmann et al. (2019) and Schlingmann et al. (2020). Also considering a possible underestimation of N_2_ emission, which would result in an overestimation of NH_3_ emissions due to our mass balance approach, our data still indicate that N_2_ emissions remained clearly smaller than NH_3_ emissions, but still represent the second most important gaseous N loss pathway.

### Dynamics of fertilizer partitioning in the plant-soil system

The high temporal resolution of this ^15^N tracing study allowed unprecedented detailed insight into the dynamics of fertilizer N fluxes. We distinguished several mechanisms and processes occurring on different time scales: (1) fertilizer input via leaching resulted in increased mineral N concentrations mainly in the top 4 cm, although N concentrations also increased somewhat in deeper soil layers (immediately after fertilization); (2) a phase marked by the rapid conversion of ammoniacal N into volatilized NH_3_, accompanied by strong nitrification and subsequent denitrification (until day 3); (3) plant N uptake of fertilizer N mainly from the top 0–4 cm of soil (until day 17), especially in the form of NO_3_^−^ after day 3; and (4) no further detectable fertilizer N dynamics between day 17–29.

Contrary to our expectations, plant N uptake of applied manure N, especially into roots, occurred at high rates as early as 1 day after fertilization and quantitatively exceeded denitrification N losses by an order of magnitude after 3 days (Fig. 3). Such high initial fertilizer N uptake by plants may be facilitated by the arbuscular mycorrhizal fungal (AMF) community, which is abundant in the extensively managed grassland of this study (Andrade-Linares et al. 2023). It should be noted that due to our sampling strategy, part of the AMF hyphae would be sampled as soil. Therefore, even more N taken up by mycorrhiza would have to be attributed to roots. The maximum plant N uptake of 100 kg N ha^−1^ reached on day 17 (of which 34 kg derived from fertilizer N) is about twice as high as in a previous study at the same site (Schlingmann et al. 2020), but similar to the N output per harvest as observed for grasslands at higher altitudes with higher SOC content (Zistl-Schlingmann et al. 2020). Despite the huge total plant N uptake, only about 1/3 of plant N was derived from recent fertilizer, leaving 2/3 to be acquired from other N sources such as biological N_2_ fixation, atmospheric deposition, and likely dominant mineralization and nitrification of soil organic N (Wang et al. 2016; Zistl-Schlingmann et al. 2020). The contribution of recent fertilizer N to plant N was even much lower (3–11%) in previous studies in comparable grasslands (Schlingmann et al. 2020).

Grassland harvesting is an important N export pathway, with exported N originating mainly from non-fertilizer N. Therefore, grassland harvesting can be a key driver of soil N mining. In this context, it is interesting to see if the application of N-rich slurry, as in our study, may prevent soil N mining. Subtracting 25 kg N (total N gaseous losses) and 0.2 kg N (leaching losses) as well as 95.6 (plant N uptake) from the N input of 97.2 kg N ha^−1^ results in a deficit of 24 kg N ha^−1^ calculated for the one month period of this study. Considering atmospheric N deposition and biological N_2_ fixation, which are estimated to be 33 and 10 kg N ha^−1^ year^−1^, respectively (Schlingmann et al. 2020) suggests a neutral N balance for two cuts and a deficit starting with three or more cuts based on our study.

Previous studies conducted at comparable grassland sites, where lower rates of cattle slurry N application were employed, reported larger N deficits ranging between 50 kg N ha^−1^ year^−1^ (2–3 fertilization/cutting cycles) and 100 kg N ha^−1^ year^−1^ (approximately 4–5 fertilization/cutting cycles). These deficits were further amplified under experimentally simulated climate change conditions (Schlingmann et al. 2020; Zistl-Schlingmann et al. 2020). Such SON mining is closely related to SOC mining as demonstrated by Wang et al. (2021) for comparable grassland systems. In this context, N mining can be mitigated either by reduced plant N output and/or when fertilizer N losses are reduced. The fertilizer N losses in this study, at 25% of the applied N, are relatively small and consistent with the findings of Schreiber et al. (2023). In contrast, Schlingmann et al. (2020) and Zistl-Schlingmann et al. (2020) reported slurry N losses ranging between 40–60% of applied N, despite all three studies adhering to similar slurry application timings. Given this large variability of slurry N losses, further studies should be targeted to identify the management, soil and climatic factors that govern slurry N fates. Particularly, improved slurry application techniques such as open slot injection or slurry acidification (Buchen-Tschiskale et al. 2023; Emmerling et al. 2020; Schreiber et al. 2023) are expected to reduce grassland N losses and SON mining.

## Conclusion

The present study constrains both N_2_O and N_2_ emissions from organic matter-rich pre-alpine grasslands after manure application based on direct in situ ^15^N gas flux measurements. However, it also highlights the associated uncertainties due to persistent methodological problems. Our results suggest that N-rich slurry application does not lead to large N_2_O losses from organic matter-rich pre-alpine grassland soils with neutral pH, at least when fertilization is followed by precipitation events. This is partly due to the reduction of N_2_O to N_2_ and partly due to the rapid uptake of N by plants, which exceeds total denitrification by an order of magnitude already 3 days after fertilization. As a result, N_2_ emissions accounted for only 3% of the added fertilizer N. The high plant N uptake, together with microbial immobilization, appears to enhance the filter capacity of the soil, preventing N leaching despite high nitrification of ammoniacal N from the fertilizer and extensive precipitation, which generally favors N leaching. Thus, plants played a pivotal role in mitigating significant denitrification N losses and leaching, which underscores the importance of N fertilization timing adjusted to grass growth patterns to reduce N losses. However, our N mass balance estimates indicate that NH_3_ losses were up to 20% of the applied N, emerging as the predominant N loss pathway. Our findings also suggest that dark chamber incubations, typically used in He soil core approaches, may severely overestimate denitrification in such grasslands due to impaired plant N uptake. The high temporal resolution of fertilizer ^15^N partitioning data in the plant-soil microbial system provided in this study is excellent for comprehensive testing of biogeochemical models, which can ultimately be used to further explore sustainable grassland management options at the regional scale.

## Supplementary information

Below is the link to the electronic supplementary material.

Supplementary file1 (DOCX 641 KB)
