# Supplementary material for: Fates of slurry-nitrogen applied to mountain grasslands: the importance of dinitrogen emissions versus plant N uptake
Source: Biol Fertil Soils. 2024 May 10;61(3):455–68. doi: 10.1007/s00374-024-01826-9 (PMC11910402; doi:10.1007/s00374-024-01826-9)
Supplement: Supplementary file 1 — Supplementary file1 (DOCX 641 KB) [file 374_2024_1826_MOESM1_ESM.docx]

**Supplementary Figures**

**Fates of slurry-nitrogen applied to mountain grasslands: the importance of dinitrogen emissions versus plant N uptake**

Michael Dannenmann^a^, Irina Yankelzon^a^, Svenja Wähling^a^, Elisabeth Ramm^a^, Mirella Schreiber^a^, Ulrike Ostler^a^, Marcus Schlingmann^a, b^, Claus Florian Stange^c^, Ralf Kiese^a^, Klaus Butterbach-Bahl^a, d^ Johannes Friedl^e^, Clemens Scheer^a^

a) Karlsruhe Institute of Technology (KIT), Institute of Meteorology and Climate Research, Atmospheric Environmental Research (IMK-IFU), Kreuzeckbahnstraße 19, 82467 Garmisch-Partenkirchen, Germany

b) Landwirtschaftliches Zentrum Baden-Württemberg (LAZBW), Grassland Division, Lehmgrubenweg 5, 88326 Aulendorf, Germany

c) Federal Institute for Geosciences and Natural Resources (BGR), Stilleweg 2, 30655 Hannover, Germany

d) Land-CRAFT, Department of Agroecology, University of Aarhus, Ole Worms Allé 3, Bld. 1171, 8000 Aarhus, Denmark

e) Department of Forest and Soil Sciences, Institute of Soil Research, University of Natural Resources and Life Sciences, Vienna, Austria

*Corresponding author: Michael Dannenmann; e-mail: [michael.dannenmann@kit.edu](mailto:michael.dannenmann@kit.edu), Phone: +49 8821 183127


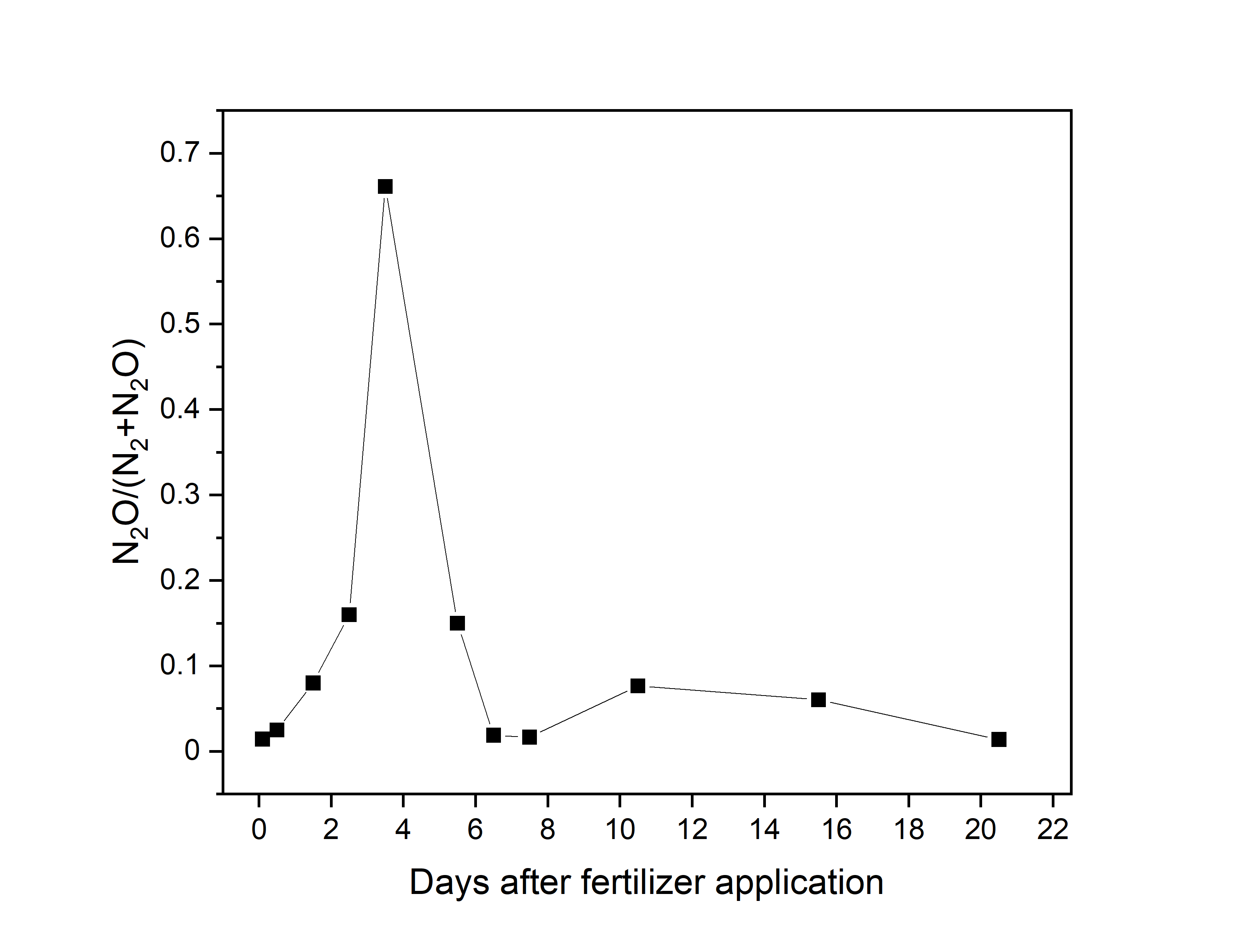


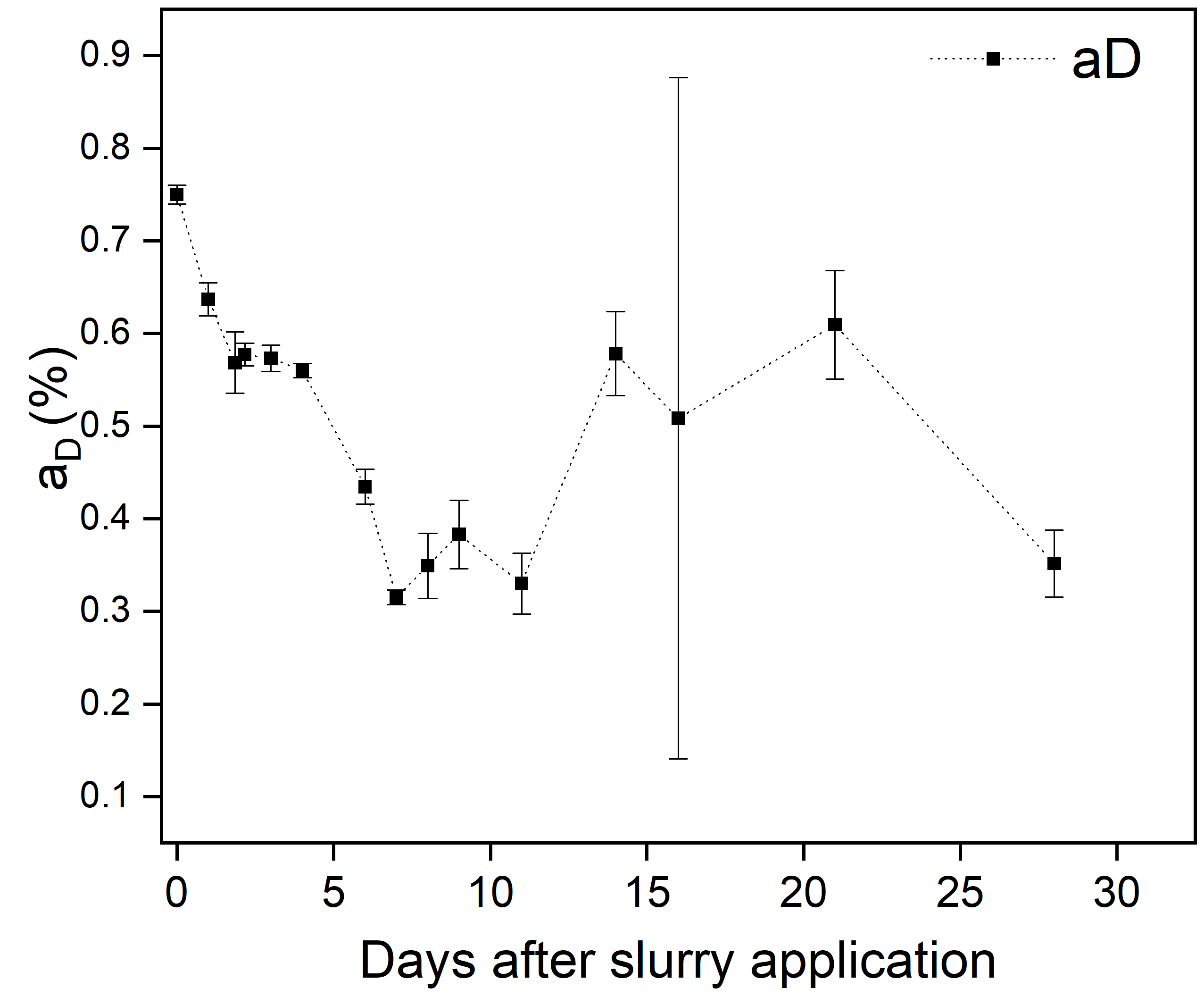


**Fig. S1** Mean ± SE (n=6) enrichment in the NO_3_^-^ pool undergoing denitrification (aD) values in % in N_2_O following the slurry application.

**Fig. S2:** The N_2_O/(N_2_ + N_2_O) emission ratio during the course of the measurements. Note that the relatively high values between days 4-6 occurred at low emissions of both N_2_O and N_2_ (see Fig. 2). The overall cumulative R_N2O_ was as low as 0.03.


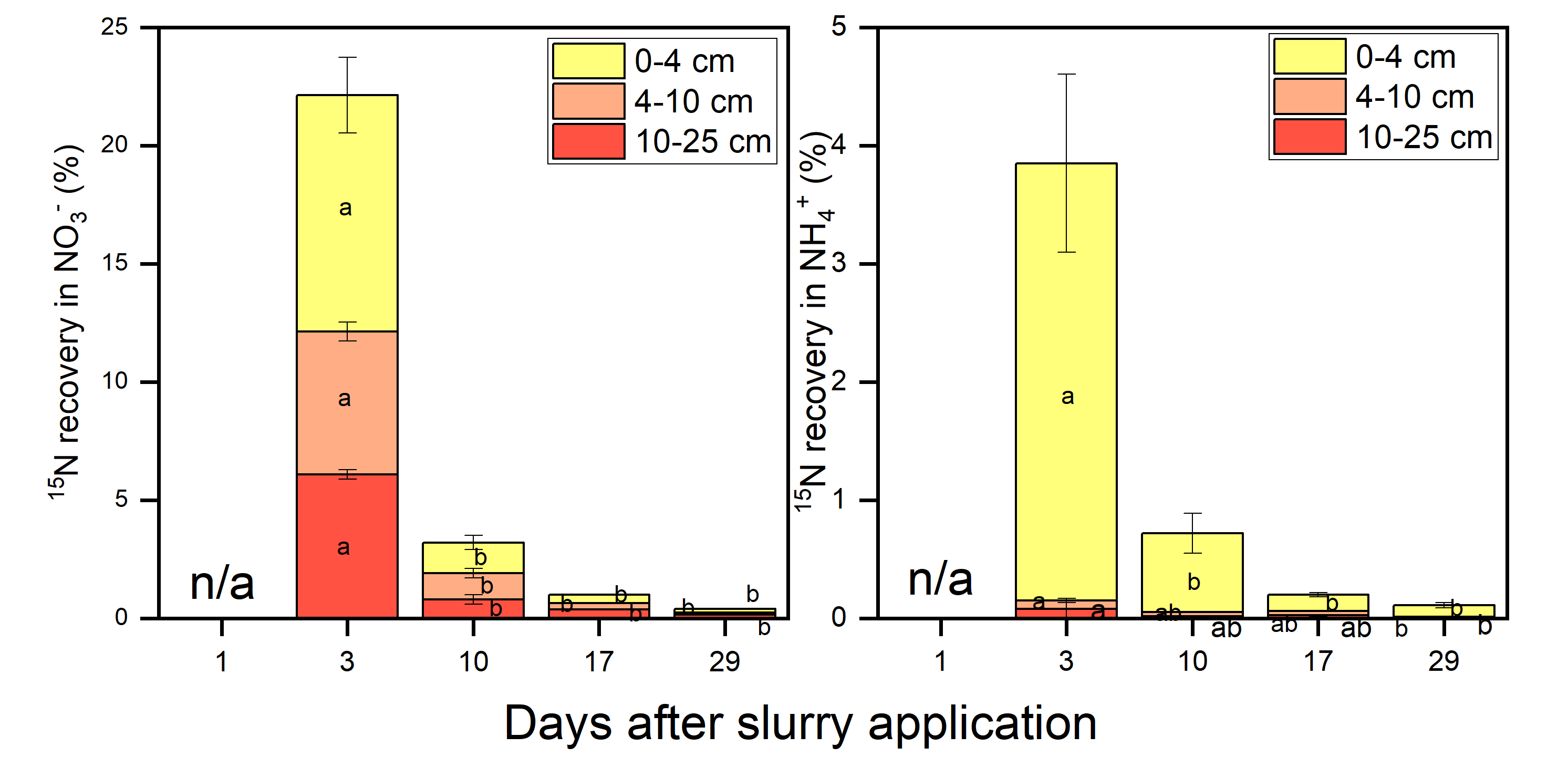


**Fig. S3** Recovery of ^15^N in inorganic soil N compounds. On the left there is ^15^N recovery in nitrate in three soil depths. On the right, there is ^15^N recovery in ammonium. The units are percent and represent the % of the total ^15^N recovered in soil. The error bars represent the standard error of the mean (n=6). The letters denote statistically significant differences within each depth on various dates (p < 0.05).

**Fig. S4** DOC concentrations in three soil layers after slurry application. The error bars represent standard error of the mean (n=6). The letters denote statistically significant differences within each depth on various dates (p < 0.01).


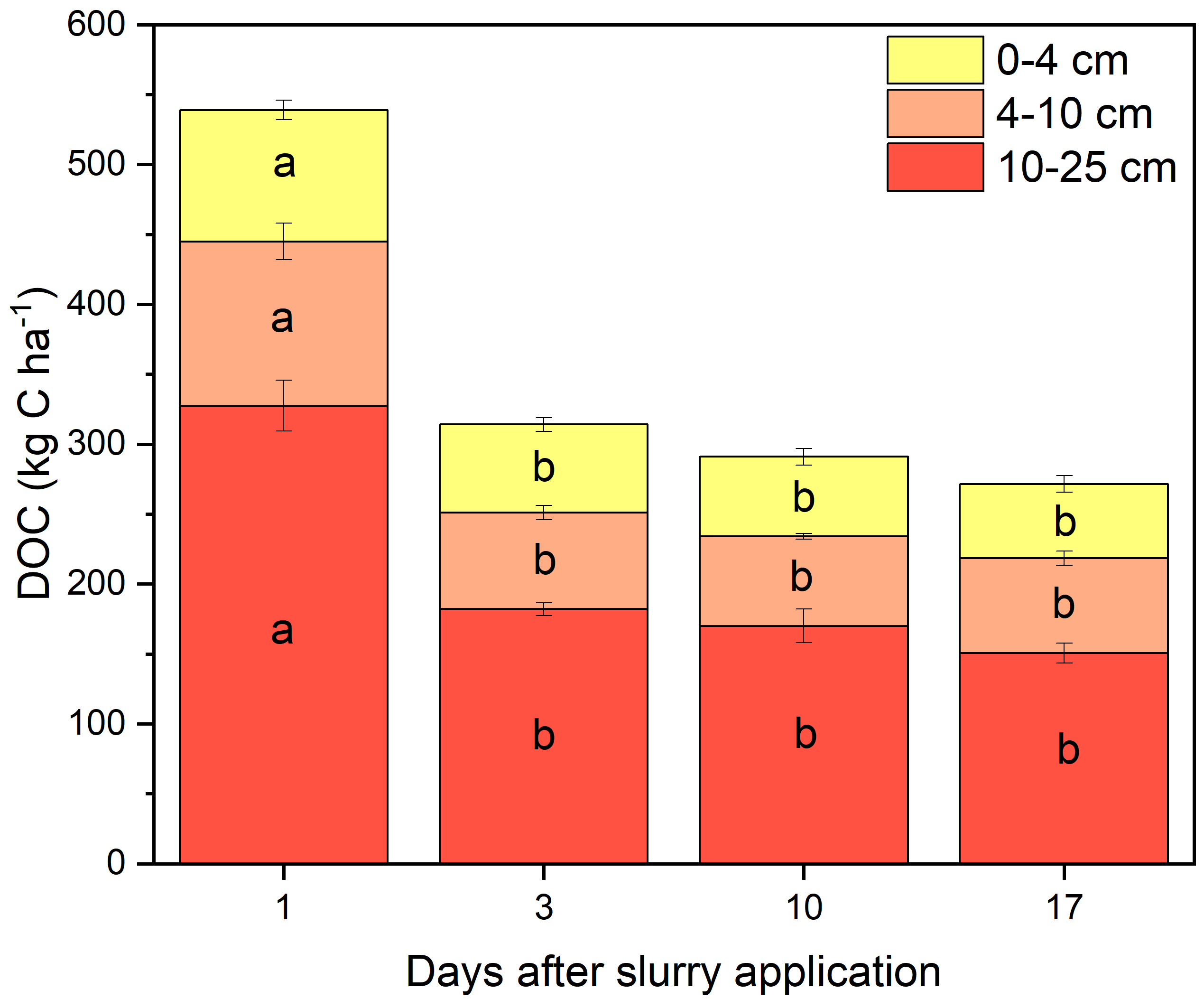


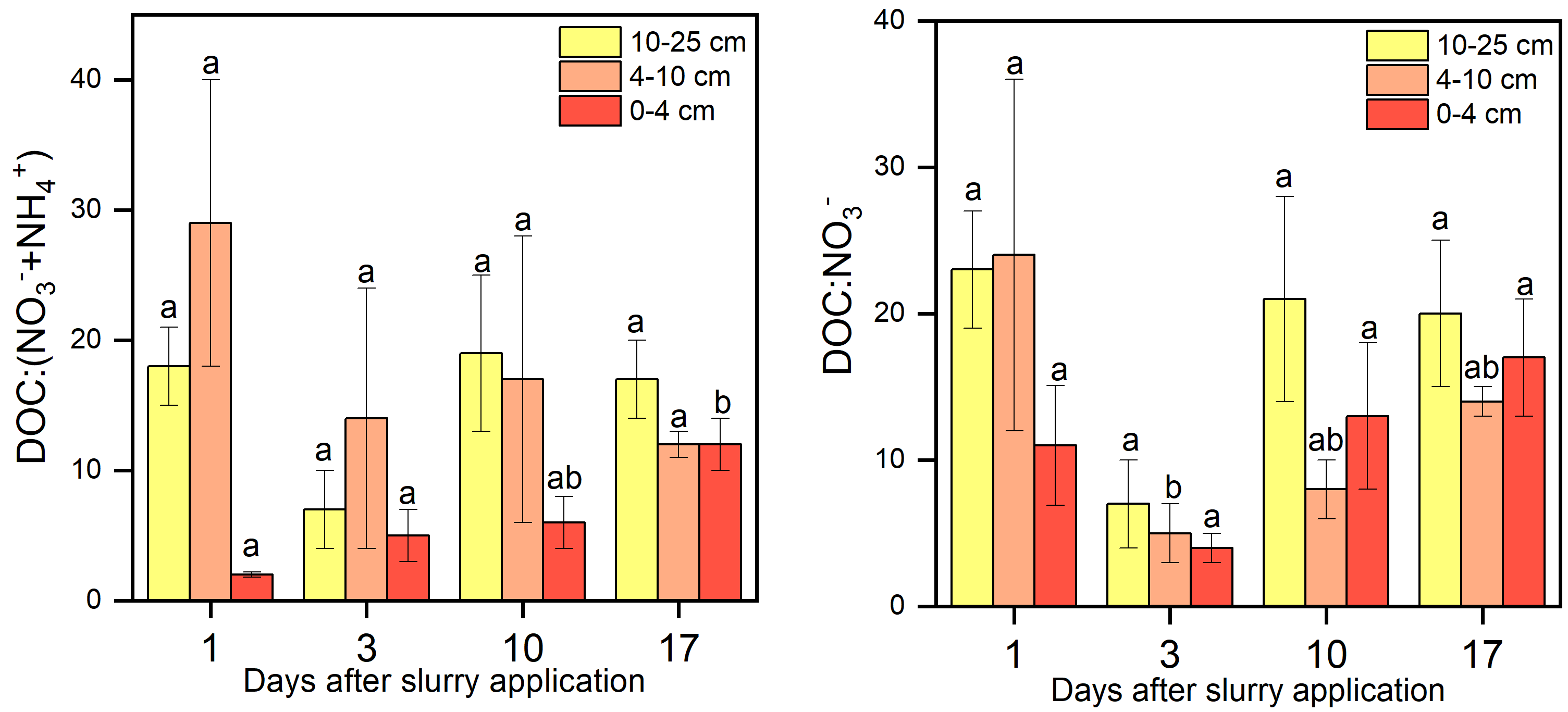


**Fig. S5** Ratio of DOC to (NO_3_^-^ + NH_4_^+^) over the experimental period following slurry application at three different depths. The letters denote statistically significant differences within each depth on various dates (p < 0.05).

**Fig. S6** Ratio of DOC to NO_3_^-^ over the experimental period following slurry application at three different depths. The letters denote statistically significant differences within each depth on various dates (p < 0.05).
